# A Case of Heterotopic Ossification in Papillary Renal Cell Carcinoma Type 2

**DOI:** 10.1155/2020/5126802

**Published:** 2020-08-14

**Authors:** Facundo Davaro, Elizabeth Davaro, Amna Qureshi, Lindsay Lombardo

**Affiliations:** ^1^Division of Urology, Department of Surgery, Saint Louis University, 3635 Vista Ave, 3rd Floor Desloge Towers, St. Louis, MO 63110, USA; ^2^Department of Pathology, Saint Louis University, 3635 Vista Ave, 4th Floor Desloge Towers, St. Louis, MO 63110, USA

## Abstract

Renal cell carcinoma (RCC) is associated with a variety of different histopathologic subtypes in which each subtype may be further subclassified. These entities carry with them unique prognoses and necessitate treatment with specific immunotherapy agents should advanced disease be uncovered. Meanwhile, aberrant physiologic processes may lead to unique histologic findings within these subtypes, further complicating management and prognostication. Heterotopic ossification within RCC is one of these rare occurrences and was once thought to have favorable prognostic implications. We report a case of a young female with papillary type 2 RCC with heterotopic ossification.

## 1. Introduction

The process of osseous metaplasia is a rare histopathologic manifestation within renal cell carcinoma (RCC) believed to be a product of ischemia, necrosis, and/or inflammation [1]. Consequently, heterotopic ossification within RCC is thought to arise in hypovascular regions within the tumor [2]. Its presence is presumably associated with a more favorable prognosis, owing to the decreased vasculature associated with this finding [1]. Meanwhile, papillary RCC (PRCC) is the second most common subtype of RCC accounting for 10-15% of all RCCs, with type 2 carrying with it a poorer prognosis [3]. The occurrence of both histopathologic findings in a single case has been an infrequently reported phenomenon in the literature [1, 4]. We report a rare case of papillary type 2 RCC with heterotopic ossification.

## 2. Case Presentation

**Figure 1:**
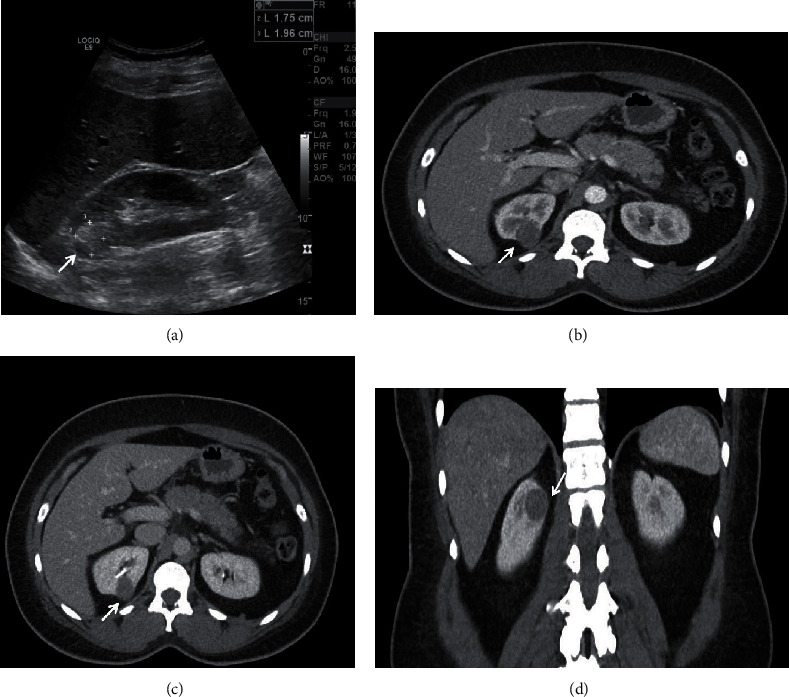
Patient preoperative imaging revealing the renal mass (white arrows). (a) Sagittal ultrasound image of the echogenic, solid renal mass measuring 1.75 cm by 1.96 cm. (b, c) Axial CT images with contrast highlighting the posterior location of the mass, its enhancing posterior wall, and nearness to the collecting system. (d) Coronal CT image of the same mass and its upper pole location. No calcifications were noted throughout the mass.

An otherwise healthy 39-year-old African-American female with a history of migraines, sickle cell trait, and morbid obesity (body mass index 37.08 kg/m^2^) presented to the emergency department with nausea, emesis, and nonbloody diarrhea. Her physical exam revealed mild abdominal tenderness to palpation on the right side. No costovertebral angle tenderness was elicited. The laboratory work indicated a normal white blood cell count and baseline renal function as evidenced by a creatinine of 0.8 mg/dL. Food poisoning was suspected due to her reporting recent consumption of an “uncooked burger”; however, a computed tomography (CT) scan without contrast was performed to rule out more serious pathology. In doing so, an incidental right renal lesion was found. She was discharged with oral ondansetron 5 mg as needed for nausea and a scheduled follow-up with urology. To further characterize the mass, a renal protocol computed tomography scan with contrast was performed prior to her outpatient follow-up. This revealed a 2.5 cm renal mass located in the posterior, upper pole of the right kidney with post contrast enhancement, suspicious for malignancy. The R.E.N.A.L. nephrometry score was calculated at 7p (Figure 1). Given her young age, the patient opted for definitive treatment after risks, benefits, and alternatives of the procedure were discussed. She subsequently underwent a right robotic partial nephrectomy. The patient's hospital course was uncomplicated, and she was discharged on postoperative day one.

**Figure 2:**
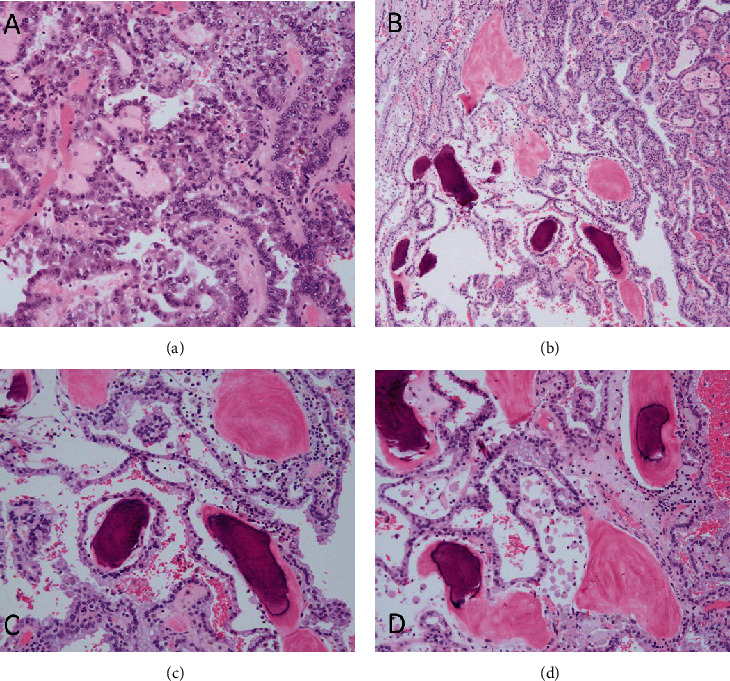
(a) Hematoxylin and eosin-stained sections representative of tumor with nuclear pseudostratification and nuclear enlargement consistent with papillary renal cell carcinoma type 2. Granular to eosinophilic cytoplasm is also seen. (b) A low power view with heterotopic ossification with intervening tumor. (c, d) On higher power, focal osteoblastic rimming is seen surrounding trabecular woven bone.

Gross pathologic examination demonstrated a well-circumscribed tumor, the cut surface of which showed a variegated yellow-tan 2.1 × 1.9 cm mass with punctate hemorrhages. The patient staged with pT1a disease. Microscopic examination confirmed a papillary renal cell carcinoma, type 2, with an associated International Society of Urologic Pathologist (ISUP) grade 2 as evidenced by Figure 2(a). Irregular deposits of bone could be seen intermixed with the tumor cells (Figures 2(b)–2(d)).

Postoperatively, the patient was instructed to return to the clinic in 2 weeks for a wound check and counseling over her diagnosis. The patient appeared well at this time with resolution of pain and increasing appetite and energy. Her 18-month follow-up imaging did not demonstrate any evidence of locoregional or distant recurrence, and her creatinine remained near her baseline at 1.0 mg/dL.

## 3. Discussion

Renal cell carcinoma has been categorized into 16 histologic subtypes by the 2016 World Health Organization (WHO) [3]. Within these subtypes, further classification occurs helping clinicians determine evidence-based management decisions and prognostication information used in patient counseling. A classic example of this substratification is seen in PRCC, as patients are labeled as having type 1 or type 2 disease. Clinically, type 1 disease is associated with superior survival outcomes relative to type 2 [5]. Microscopically, type 1 is characterized by having simple cuboidal epithelium distinguishing it from type 2 which demonstrates true cellular pseudostratification with eosinophilic cytoplasm [3]. In a histopathologic review of PRCC at their institution, Warrick et al. retrospectively examined 56 cases of PRCC attempting to elucidate histomorphologic features that distinguish the two papillary subtypes from one another. The presence of psammoma bodies, foamy macrophages, and eosinophilic cytoplasm along with several other histologic features was catalogued within the tumors. They found that although classic features of type 1 and type 2 tended to cluster together, no singular characteristics were able to reliably differentiate one from the other [6].

Beyond the classic differences in histologic architecture, other rare, nuanced microscopic findings have unclear implications and may complicate this substratification even further. Histopathologic findings such as bone marrow within a RCC or solitary fibrous tumor of the kidney have been reported [7, 8]. Heterotopic ossification is another, even rarer microscopic finding within RCC. It is the formation of extraskeletal bone in muscle or soft tissue as defined by Meyers et al. [9]. Several case reports discussing the finding have been published in varying types of RCC; however, to date, there have been few case reports describing this histopathologic finding in type 2 PRCC [1, 4].

Investigations into molecular pathways contributing to heterotopic ossification have been undertaken in order to elucidate the prognostic implications of its presence. For example, hypoxia-inducible factor and mammalian target of rapamycin signaling pathways are involved in heterotopic ossification [9]. These are known aberrant pathways in RCC that have been targeted with immunotherapy for treatment. In vitro experiments inhibiting hypoxia-inducible factor have been conducted revealing an increase in the expression of bone morphogenetic proteins, a family of proteins involved in osseous metaplasia [10]. In their research, Wang et al. corroborate the dogma that heterotopic ossification is a process that predominantly occurs in hypoxic environments. Furthermore, when they performed in vivo stimulation of bone morphogenetic protein-2 in certain mouse cell lines with RCC, they noted an arrest in tumor growth. Unfortunately, this response was not uniform throughout all mouse cell lines indicating the need for further research in the underlying signaling pathways [11].

Given the significant rarity of this histopathologic occurrence, any conclusions about the etiology of heterotopic ossification in type 2 PRCC, or RCC as a whole, are purely conjecture.

## 4. Conclusion

Heterotopic ossification is an atypical phenomenon infrequently encountered in RCC. Although presumed to have favorable prognostic implications, its rarity makes it difficult to establish any true associations.

## Abbreviations

RCC: Renal cell carcinoma
PRCC: Papillary renal cell carcinoma
R.E.N.A.L.: Radius, endophytic/exophytic, nearness, anterior/posterior, location
ISUP: International Society of Urologic Pathologists
WHO: World Health Organization.
